# Multi-nuclear sodium, diffusion, and perfusion MRI in human gliomas

**DOI:** 10.1007/s11060-023-04363-x

**Published:** 2023-06-09

**Authors:** Nicholas S. Cho, Francesco Sanvito, Shruti Thakuria, Chencai Wang, Akifumi Hagiwara, Raksha Nagaraj, Sonoko Oshima, Alfredo L. Lopez Kolkovsky, Jianwen Lu, Catalina Raymond, Linda M. Liau, Richard G. Everson, Kunal S. Patel, Won Kim, Isaac Yang, Marvin Bergsneider, Phioanh L. Nghiemphu, Albert Lai, David A. Nathanson, Timothy F. Cloughesy, Benjamin M. Ellingson

**Affiliations:** 1 UCLA Brain Tumor Imaging Laboratory (BTIL), Center for Computer Vision and Imaging Biomarkers, University of California, Los Angeles, Los Angeles, CA USA; 2 Department of Radiological Sciences, David Geffen School of Medicine, University of California, Los Angeles, Los Angeles, CA USA; 3 Department of Bioengineering, Henry Samueli School of Engineering and Applied Science, University of California, Los Angeles, Los Angeles, CA USA; 4 Medical Scientist Training Program, David Geffen School of Medicine, University of California, Los Angeles, Los Angeles, CA USA; 5 Department of Radiology, Juntendo University School of Medicine, Tokyo, Japan; 6 NMR Laboratory, Neuromuscular Investigation Center, Institute of Myology, Paris, France; 7 Department of Neurosurgery, David Geffen School of Medicine, University of California, Los Angeles, Los Angeles, CA USA; 8 UCLA Neuro-Oncology Program, David Geffen School of Medicine, University of California, Los Angeles, Los Angeles, CA USA; 9 Department of Neurology, David Geffen School of Medicine, University of California, Los Angeles, Los Angeles, CA USA; 10 Department of Molecular and Medical Pharmacology, David Geffen School of Medicine, University of California, Los Angeles, Los Angeles, CA USA; 11 Department of Psychiatry and Biobehavioral Sciences, David Geffen School of Medicine, University of California, Los Angeles, Los Angeles, CA USA; 12 UCLA Brain Tumor Imaging Laboratory (BTIL) Professor of Radiology, Psychiatry, and Neurosurgery Departments of Radiological Sciences, Psychiatry, and Neurosurgery David Geffen School of Medicine, University of California, Los Angeles, 924 Westwood Blvd., Suite 615, Los Angeles, CA 90024 USA

**Keywords:** Glioma, Sodium MRI, Diffusion MRI, Dynamic susceptibility contrast perfusion MRI, Multinuclear MRI, Glioblastoma

## Abstract

**Purpose:**

There is limited knowledge about the associations between sodium and proton MRI measurements in brain tumors. The purpose of this study was to quantify intra- and intertumoral correlations between sodium, diffusion, and perfusion MRI in human gliomas.

**Methods:**

Twenty glioma patients were prospectively studied on a 3T MRI system with multinuclear capabilities. Three mutually exclusive tumor volumes of interest (VOIs) were segmented: contrast-enhancing tumor (CET), T2/FLAIR hyperintense non-enhancing tumor (NET), and necrosis. Median and voxel-wise associations between apparent diffusion coefficient (ADC), normalized relative cerebral blood volume (nrCBV), and normalized sodium measurements were quantified for each VOI.

**Results:**

Both relative sodium concentration and ADC were significantly higher in areas of necrosis compared to NET (*P* = 0.003 and *P* = 0.008, respectively) and CET (*P* = 0.02 and *P* = 0.02). Sodium concentration was higher in CET compared to NET (*P* = 0.04). Sodium and ADC were higher in treated compared to treatment-naïve gliomas within NET (*P* = 0.006 and *P* = 0.01, respectively), and ADC was elevated in CET (*P* = 0.03). Median ADC and sodium concentration were positively correlated across patients in NET (r = 0.77, *P* < 0.0001) and CET (r = 0.84, *P* < 0.0001), but not in areas of necrosis (r = 0.45, *P* = 0.12). Median nrCBV and sodium concentration were negatively correlated across patients in areas of NET (r=-0.63, *P* = 0.003). Similar associations were observed when examining voxel-wise correlations within VOIs.

**Conclusion:**

Sodium MRI is positively correlated with proton diffusion MRI measurements in gliomas, likely reflecting extracellular water. Unique areas of multinuclear MRI contrast may be useful in future studies to understand the chemistry of the tumor microenvironment.

**Supplementary Information:**

The online version contains supplementary material available at 10.1007/s11060-023-04363-x.

## Introduction

Proton (^1^H) magnetic resonance imaging (MRI) is the gold standard for diagnosis and management of human gliomas. Gliomas are a heterogeneous group of tumors that account for 27% of all primary central nervous system (CNS) tumors [1] and are considered uniformly fatal even with aggressive treatment [2, 3]. Contrast enhancement on post-contrast, T1-weighted proton MRI can be used to define the contrast-enhancing component of the tumor (CET), which contains the most aggressive high-grade features of the tumor [4, 5], while T2-weighted proton images including fluid-attenuated inversion recovery (FLAIR) sequences are useful for defining non-enhancing tumor (NET), which contains the bulk of the tumor in lower grade gliomas and a combination of infiltrative glioma cells and edema in higher grade tumors [6].

In addition to these standard anatomic proton MRI sequences that are used to isolate areas of concern, advanced physiologic imaging techniques including diffusion and perfusion MRI are often used to explore cellularity and vascularity, respectively, within these regions. The apparent diffusion coefficient (ADC) measured using proton diffusion weighted imaging (DWI) has been shown to be sensitive to cell density and proliferation [7], and ADC values are reported to differ between treated, recurrent tumors and radiation necrosis or pseudoprogression [8]. Additionally, the extracellular space and volume fraction are increased in brain tumors compared to healthy brain tissue as infiltrating glioma cells deposit extracellular matrix components along with extravasation of fluid from leaky vasculature [9, 10], which increase ADC [11]. Dynamic susceptibility contrast (DSC) perfusion MRI is also used often in human gliomas to isolate areas of high vascular density [12], identify highly aggressive areas of the tumor undergoing angiogenesis [6], and may also be useful in differentiating recurrent tumor from pseudoprogression [13].

While proton MRI is commonly used in clinical care given the abundance of water protons and the intrinsically high proton magnetic moment, other nuclei, including sodium (^23^Na), may complement proton MRI [14, 15] given the importance of sodium homeostasis for healthy tissue and gliomas. Intracellular (10–15 mM) and extracellular sodium concentrations (140–150 mM) are tightly regulated by well-known mechanisms, including the Na^+^/K^+^-ATPase and Na^+^/H^+^-exchangers (NHEs) [15, 16]. In gliomas, NHE1 is important to maintain an intracellularly alkaline environment [17] and is implicated in resistance to temozolomide chemotherapy [18]. Matched recurrent gliomas have higher expression of NHE1 compared to primary gliomas, which is also associated with reduced overall survival [19]. However, several intrinsic and technical challenges have limited clinical sodium MRI applications. For example, while sodium is the second-most abundant MR-detectable nucleus in the body after ^1^H, its intrinsic MR sensitivity is nearly 1/10,000th that of proton, has a concentration of below 0.1% that of water protons, and presents short biexponential signal decay times in tissue [14, 15].

Early sodium MRI studies have shown elevated sodium in brain tumors compared to normal brain [20, 21], which has been associated from a potential combination of increased intracellular sodium from altered sodium homeostasis in malignancy [22] and from increased extracellular volume fraction [21]. Sodium MR contrast also demonstrates intra-tumor heterogeneity between tumor subregions, with necrotic areas reportedly exhibiting higher sodium signal intensity than CET and NET [23]. Furthermore, total sodium MR signal has been shown to be higher in human isocitrate dehydrogenase (IDH)-mutant (IDH-mut) gliomas compared to IDH-wild-type (IDH-wt) gliomas prior to chemoradiation [23, 24]. However, other studies reported that IDH-wt gliomas exhibit higher sodium signal than IDH-mut gliomas when imaged with advanced sodium MRI acquisition techniques, which are believed to be more sensitive to intracellular sodium by targeting sodium ions with restricted mobility [24, 25]. Sodium MR signal also increases after radiosurgery in brain metastases [26] and vestibular schwannoma [27], and sophisticated sodium MR-derived metrics including tumor cell volume fraction can demonstrate changes in glioblastomas after chemoradiation [28], suggesting sodium MRI may provide value for therapeutic response assessment.

However, no studies have examined whether similar information to sodium MRI is already available using common proton MRI techniques in human gliomas. Preclinical data suggest sodium concentration and proton ADC both increase after chemotherapy [29, 30], but to our knowledge, there remains no study assessing the potential association between sodium and proton MRI, including diffusion and perfusion MRI, in human gliomas. For example, in an early study utilizing sodium MRI in human gliomas, Ouwerkerk et al. speculated that increased neoangiogenesis may contribute to elevated sodium MR contrast in gliomas [21], so studies combining sodium and perfusion MRI would be valuable to explore this potential association. Furthermore, better characterizing the potential relationships between sodium and proton MRI measurements within tumor subregions may provide new insights into brain tumor biology.

The purpose of this prospective study was to utilize sodium and quantitative proton MRI to investigate human gliomas. We hypothesized that sodium would be highest in necrotic regions compared to CET and NET regions, and that sodium would be increased in treated tumors compared to treatment-naïve tumors. We also theorized that sodium concentration would be positively correlated with ADC given the associations of ADC with extracellular space and positively correlated with nrCBV given the associations of nrCBV and tumor malignancy.

## Methods

### Patient selection

This prospective study was performed in compliance with the Health Insurance Portability and Accountability Act and approved by our institutional review board (IRB# 21–000514). All patients provided written informed consent. Twenty glioma patients were studied, and patient data are summarized in Table 1 (see Online Resource Supplementary Table 1 for detailed patient information). Gliomas were classified based on the 2021 World Health Organization classification of CNS tumors [31]. IDH1/2 mutation status was determined using immunohistochemistry and genomic sequencing analysis [32]. 1p/19q codeletion status was determined using fluorescence in situ hybridization. Most of the study population involved IDH-wt GBM (80%) and previously-treated tumors (75%).

**Table 1:** Patient data

Characteristics	Patients
**Average Age ± SD (Years)**	49 ± 13
***Sex***	
Male	14
Female	6
***IDH Status***	
Wild-type	16
Mutant	4
***Tumor Type***	
Grade 4 Glioblastoma	16
Grade 4 Astrocytoma	1
Grade 3 Astrocytoma	1
Grade 2 Oligodendroglioma	2
***Treatment Status***	
Treatment-naïve	5
Treated	15

### Image acquisition and processing

Patient scans were conducted between October 2021 and February 2023. Imaging was performed on a 3T Siemens Prisma scanner (Siemens Healthcare; Erlangen, Germany). Proton and sodium scans were conducted during the same session using a dual-tuned head volume coil (16-channel ^1^H/1-channel ^23^Na; RAPID MR International; Columbus, OH). Anatomical pre-/post-contrast high-resolution T1-weighted (1 × 1 × 1 mm isometric), T2-weighted, T2/FLAIR, and DWI images were obtained according to the international standardized brain tumor imaging protocol [33]. Voxel-wise T1-weighted subtraction maps were created from the post- and pre-contrast T1-weighted scans [4]. ADC maps were created from the DWI scans with *b*-values of 0 and 1000 s/mm^2^.

Multi-echo DSC perfusion MRI was acquired as described previously [12]. Normalized rCBV (nrCBV) maps were calculated by first motion-correcting the time-series data (*mcflirt*; Functional Magnetic Resonance Imaging of the Brain Software Library; Oxford, England), then utilizing a bidirectional contrast agent leakage correction algorithm [34] followed by normalizing the rCBV values to the mean rCBV values of the contralateral normal appearing white matter (NAWM) using 3 spherical volumes of interest (VOIs) in the centrum semiovale [35].

Sodium MRI was performed using a 3D spoiled gradient echo sequence optimized for short TE measurements with parameters: TE/TR = 2.39/10.52 ms, 5.5 mm isotropic resolution, 264 × 264 × 264 mm^3^ FOV, 39.8^o^ flip angle, 80 Hz/pixel bandwidth, 26 averages, and 10.5 min scan time. Sodium images were normalized to the mean sodium MRI signal intensity of a VOI in the vitreous humor as done in a prior study [36].

### Tumor imaging analysis

All images were registered to the T1 post-contrast scan using rigid-body registration (*tkregister2*; Freesurfer; Massachusetts General Hospital, Harvard Medical School | *flirt*; Functional Magnetic Resonance Imaging of the Brain Software Library; Oxford, England). Three mutually exclusive volumes of interest (VOIs) within the tumor were segmented: (1) contrast-enhancing tumor (CET) utilizing T1 subtraction maps; (2) suspected macroscopic, central necrosis as defined by regions of hypointensity on T1-weighted post-contrast images surrounded by contrast-enhancement; and (3) suspected non-enhancing tumor (NET) as defined by T2/FLAIR hyperintense tumor excluding CET and necrosis. For two patients, only a NET VOI was segmented because there were no areas of contrast enhancement or central necrosis. Only central necrotic VOIs larger than 0.1 cm^3^ were included to minimize potential partial volume effects. For one intraventricular case, only the nodular CET portions outside the ventricles were used for analysis to mitigate potential cerebrospinal fluid contamination. All tumor subregion segmentations were refined utilizing a semi-automated thresholding method involving the Analysis of Functional NeuroImages (AFNI) software (NIMH Scientific and Statistical Computing Core; Bethesda, MD, USA; https://afni.nimh.nih.gov) [37]. A team of trained lab members performed the initial tumor VOI segmentations, and all final VOIs were inspected by two neuroradiologists with 6 years (FS) and 11 years (SO) of experience in neuroimaging analysis.

As a result, all 20 patients were included for NET analyses, 18 patients were included for CET analyses, and 13 patients were included for tumor subregion analysis of NET, CET, and necrosis (Online Resource Supplementary Table 1). A single-slice NAWM region of interest (ROI) was also segmented in the centrum semiovale. Median and voxel-wise normalized sodium, nrCBV, and ADC values were obtained for each VOI and ROI.

### Statistical analysis

Statistical analyses were performed using GraphPad Prism software (Version 8.4 GraphPad Software, San Diego, California). Non-parametric tests were used for comparisons involving four or less cases. Voxel-wise correlations were assessed using the Pearson correlation. For all other comparisons, the Shapiro-Wilk test for normality was used to determine whether to apply non-parametric or parametric statistical methods. Tumor subregion differences in sodium, ADC, and nrCBV were assessed using the Repeated-Measures ANOVA test with post-hoc Tukey’s multiple comparisons test or Friedman test with post-hoc Dunn’s multiple comparisons test. Differences in MRI metrics based on treatment status were assessed using either the Student’s t-test or Mann-Whitney test. Correlations of median tumor subregion metrics were assessed using either the Pearson or Spearman correlation. Pearson correlation coefficients (r) of significant voxel-wise correlations were compared to a theoretical value of r = 0 using the one-sample t-test for group assessment of voxel-wise correlations. Significance level was set to α = 0.05. All boxplots display the median with interquartile range.

## Results

**Fig. 1:**
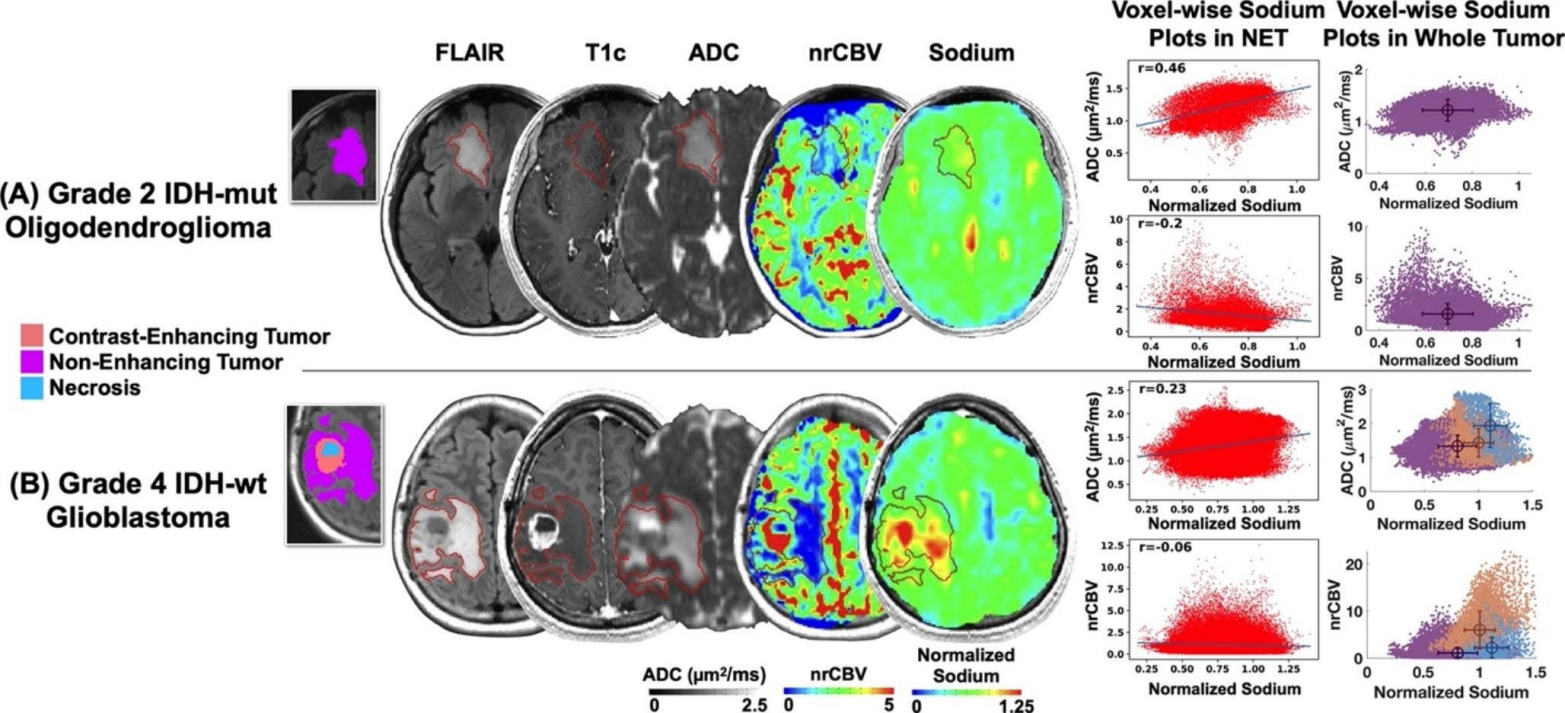
Representative cases of sodium, diffusion, and perfusion MRI associations in gliomas. Tumor volumes-of-interest were segmented as contrast-enhancing tumor (pink), non-enhancing tumor (purple), and necrosis (cyan). Patient 1 (**A**) had a newly-diagnosed grade 2 IDH-mutant oligodendroglioma. Patient 2 (**B**) had a recurrent grade 4 IDH-wild-type glioblastoma with sodium highest in necrotic regions followed by CET and NET. Both patients exhibited positive voxel-wise associations of sodium & ADC and negative voxel-wise correlations of sodium & nrCBV in NET (*P* < 0.0001 for all four correlations). There were also regions of sodium hotspots not observed on other proton MRI scans. nrCBV = normalized relative cerebral blood volume; ADC = apparent diffusion coefficient; NET = non-enhancing tumor

Two representative cases are shown in Fig. 1. The first patient was a newly-diagnosed non-enhancing IDH-mut oligodendroglioma and demonstrated moderate normalized sodium signal intensity in areas of NET (Fig. 1A), while the second patient had a recurrent, contrast enhancing IDH-wt glioblastoma and demonstrated highest sodium in necrosis followed by CET and then NET (Fig. 1B). Both patients exhibited a significant positive voxel-wise correlation between sodium and ADC, as well as a significant negative voxel-wise correlation between sodium and nrCBV within areas of NET.

**Fig. 2:**
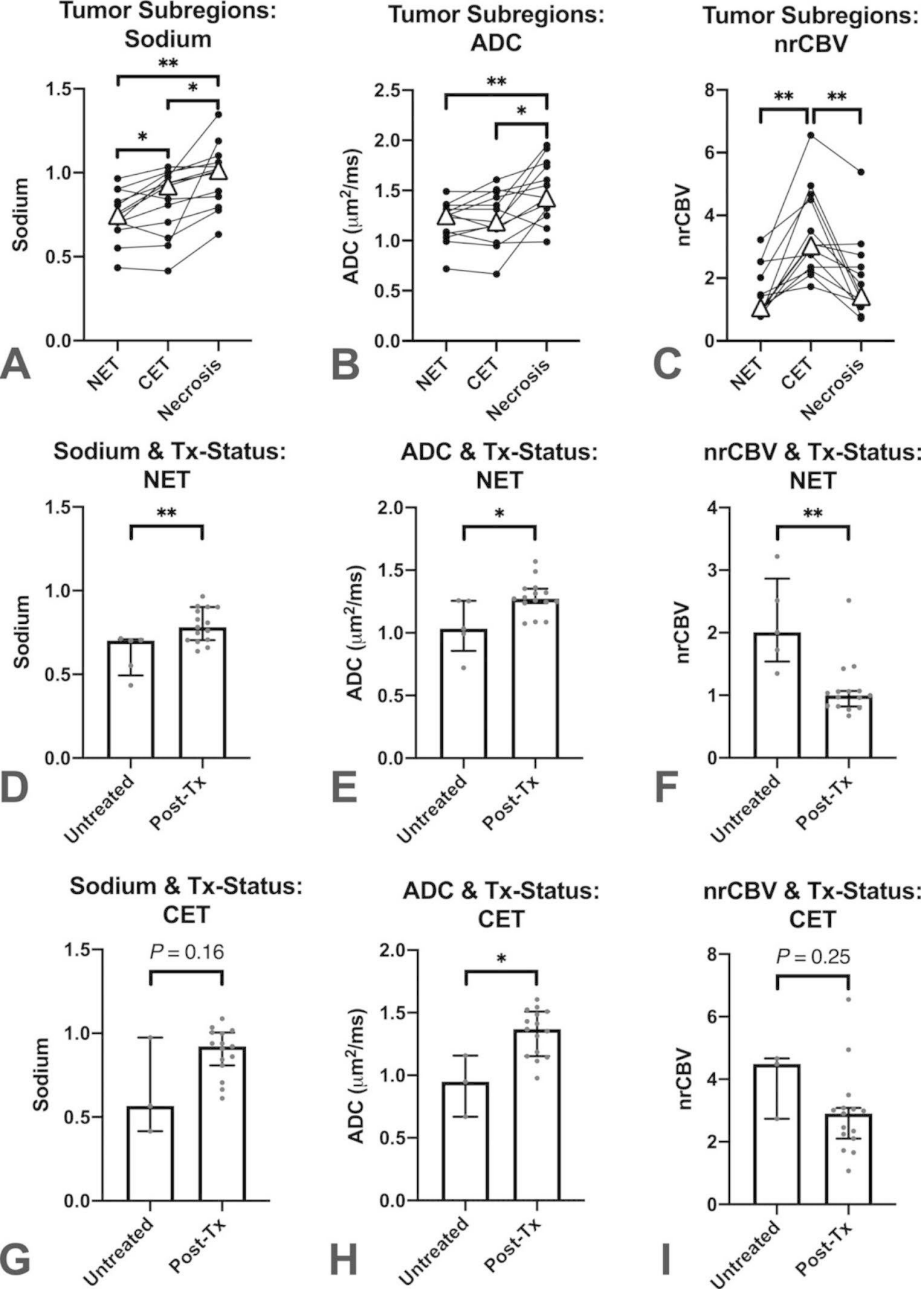
Sodium, ADC, and nrCBV differences based on tumor subregions and treatment status. Sodium was significantly highest in necrosis compared to other tumor subregions (*P* = 0.02 compared to CET and *P* = 0.003 compared to NET) and was higher in CET compared to NET (*P* = 0.04) **(A)**. ADC was highest in necrosis compared to other tumor subregions (*P* = 0.02 compared to CET and *P* = 0.008 compared to NET) but there was no significant difference in sodium level between CET and NET (*P* = 0.63) **(B)**. nrCBV was significantly higher in CET compared to NET (*P* = 0.001) and necrosis (*P* = 0.0098) **(C).** In NET, treatment-naïve tumors had a significantly lower sodium level (*P* = 0.006) **(D)**, lower ADC (*P* = 0.01) **(E)**, and higher nrCBV (*P* = 0.003) **(F)** compared to treated tumors. In CET, treatment-naïve tumors had no difference in sodium (*P* = 0.16) **(G)**, significantly lower ADC (*P* = 0.03) **(H)**, and no difference in nrCBV (*P* = 0.25) **(I)** compared to treated tumors. NET = non-enhancing tumor; CET = contrast-enhancing tumor; ADC = apparent diffusion coefficient; nrCBV = normalized relative cerebral blood volume; Tx-Status = treatment status; Post-Tx = post-treatment; △ indicates median; * indicates *P* < 0.05; ** indicates *P* < 0.01

Results show differences in sodium (*P* = 0.0007), ADC (*P* = 0.002), and nrCBV (*P* = 0.0008) across all anatomically distinct tumor subregions. In post-hoc analyses, sodium was highest in areas of central necrosis and slightly higher in areas of CET compared with NET (Fig. 2A; mean ± standard deviation = 0.98±0.19 (necrosis), 0.83±0.20 (CET), 0.74±0.15 (NET); *P* = 0.02 for necrosis vs. CET; *P* = 0.003 for necrosis vs. NET; *P* = 0.04 for CET vs. NET). ADC was also highest in areas of macroscopic necrosis but there was no significant difference in ADC measurements between CET and NET (Fig. 2B; 1.50±0.30 µm^2^/ms (necrosis), 1.22±0.26 µm^2^/ms (CET), 1.19±0.20 µm^2^/ms (NET); *P* = 0.02 for necrosis vs. CET; *P* = 0.008 for necrosis vs. NET; *P* = 0.63 for CET vs. NET). In contrast to sodium and ADC measurements, nrCBV was highest in areas of CET but no difference in nrCBV was observed between areas of necrosis and NET (Fig. 2C; 1.94±1.26 (necrosis), 3.41±1.38 (CET), 1.50±0.80 (NET); *P* = 0.001 for CET vs. NET; *P* = 0.0098 for CET vs. necrosis; *P* > 0.99 for necrosis vs. NET). The differences of sodium, ADC, and nrCBV between CET and NET remained consistent when including the subset of patients without obvious macroscopic necrosis (**Online Resource Supplementary Fig.** 1).

Sodium was significantly higher in previously-treated compared to treatment-naïve tumors, particularly in areas of suspected NET (Fig. 2D; 0.79±0.10 (treated), 0.62±0.12 (treatment-naïve), *P* = 0.006). Areas of CET tended to have slightly higher sodium in treated tumors, but this was not statistically significant (Fig. 2G; 0.89±0.14 (treated), 0.65±0.29 (treatment-naïve), *P* = 0.16). Treated tumors also had significantly higher ADC in areas of suspected NET and areas of CET compared to treatment-naïve tumors (Fig. 2E, H; NET: 1.28±0.14 (treated), 1.05±0.22 (treatment-naïve), *P* = 0.01; CET: 1.34±0.18 (treated), 0.93±0.25 (treatment-naïve), *P* = 0.03). Conversely, areas of suspected NET had significantly lower nrCBV in treated compared with treatment-naïve tumors (Fig. 2F; 1.10±0.45 (treated), 2.16±0.73 (treatment-naïve), *P* = 0.003); however, nrCBV did not differ between treated and treatment-naïve tumors in areas of CET (Fig. 2I; 2.91±1.36 (treated), 3.96±1.07 (treatment-naïve), *P* = 0.25).

**Fig. 3:**
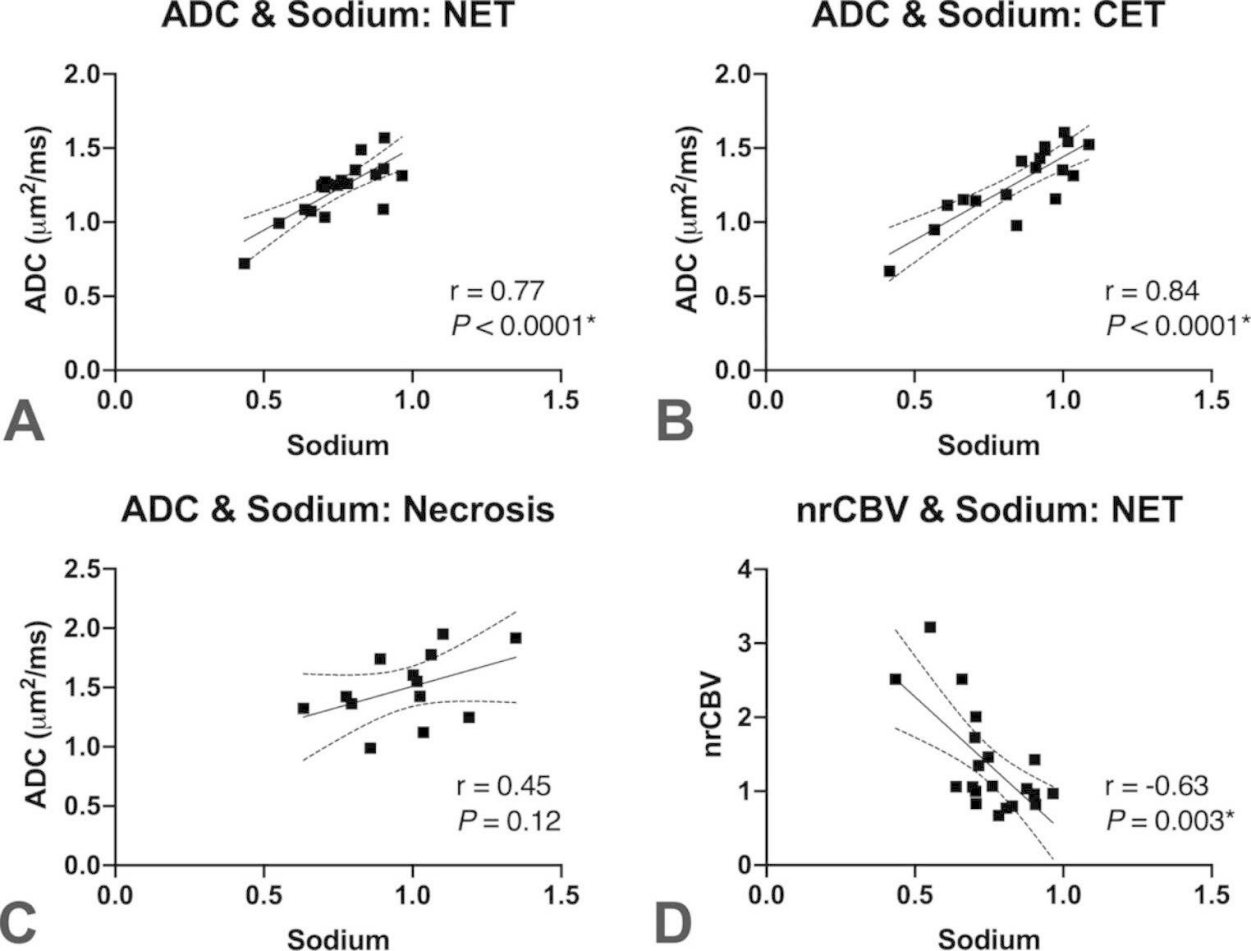
Sodium correlations of median values in tumor subregions. There was a significantly positive correlation between ADC and sodium level in NET (r = 0.77, *P* < 0.0001) **(A)** and CET (r = 0.84, *P* < 0.0001) **(B)** but no significant relationship in necrosis (r = 0.45, *P* = 0.12) **(C)**. There was also a significant negative correlation between nrCBV and sodium level in NET (r  =  -0.63, *P* = 0.003) **(D)**. NET = non-enhancing tumor; CET = contrast-enhancing tumor; ADC = apparent diffusion coefficient; nrCBV = normalized relative cerebral blood volume

**Fig. 4:**
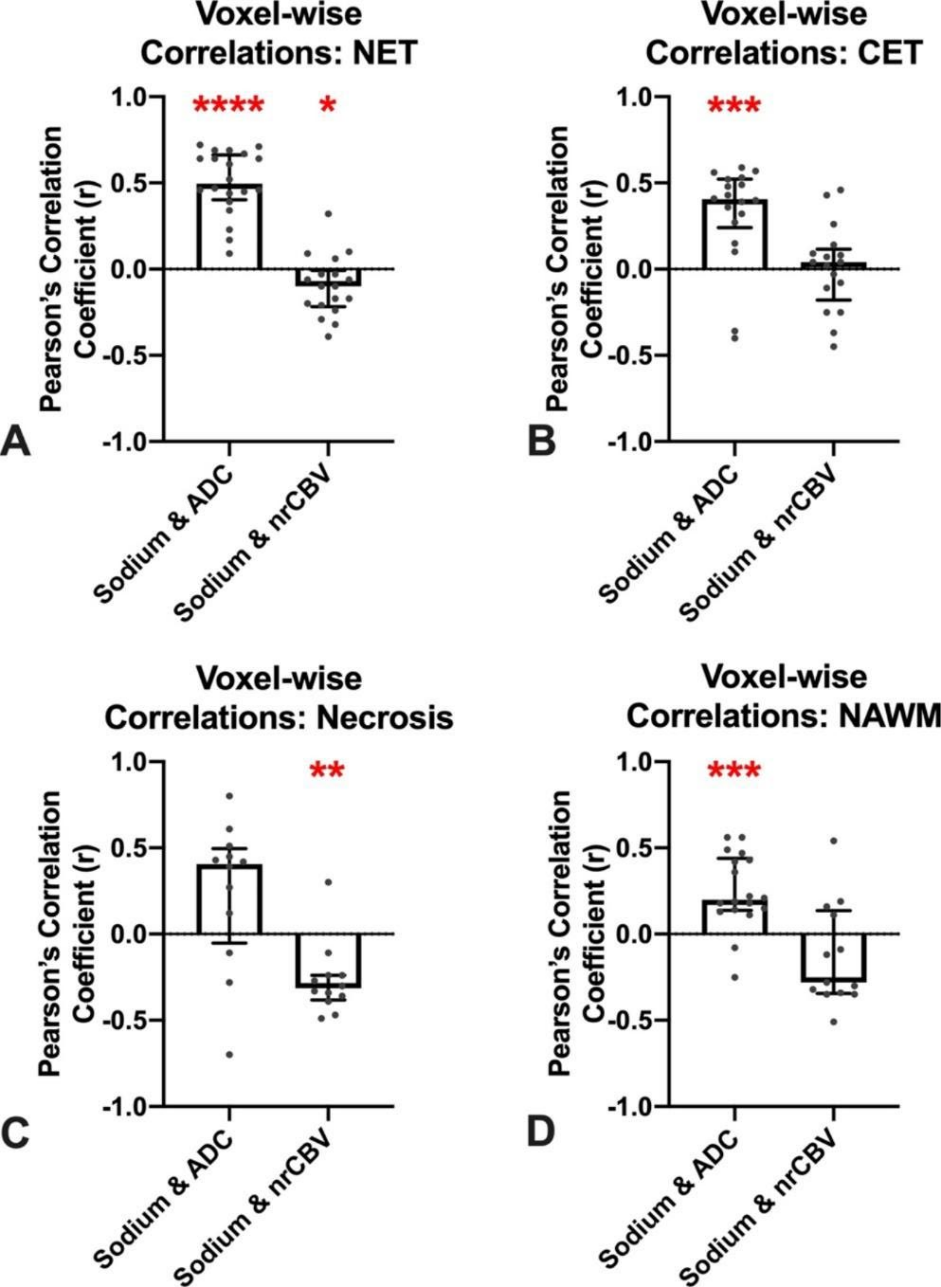
Voxel-wise sodium correlations in tumor subregions. Each dot represents the significant voxel-wise Pearson correlation value for one patient. Voxel-wise correlations were significantly different from zero and overall positive between sodium and ADC in NET (*P* < 0.0001) **(A)** and CET (*P* = 0.0008) **(B).** Voxel-wise correlations were significantly different from zero and overall negative between sodium and nrCBV in NET (*P* = 0.03) **(A)** and necrosis (*P* = 0.005) **(C)** but not in CET (*P* = 0.93) **(B)**. In exploratory analyses, voxel-wise correlations in NAWM of ADC and sodium were significantly different from zero and overall positive (*P* = 0.0002) and no significant correlation between sodium and nrCBV (*P* = 0.1) **(D)**. NET = non-enhancing tumor; CET = contrast-enhancing tumor; ADC = apparent diffusion coefficient; nrCBV = normalized relative cerebral blood volume; NAWM = normal-appearing white matter; * indicates *P* < 0.05; ** indicates *P* < 0.01; *** indicates *P* < 0.001; **** indicates *P* < 0.0001

In suspected NET and CET, median sodium and ADC were positively correlated across all patients evaluated (Fig. 3A-B NET: r = 0.77, *P* < 0.0001; CET: r = 0.84, *P* < 0.001), but not when examining areas of macroscopic necrosis (Fig. 3C; r = 0.45, *P* = 0.12). Conversely, median sodium in areas of suspected NET was negatively correlated with nrCBV across patients (Fig. 3D; r  =  -0.63, *P* = 0.003), but not in areas of CET (*P* = 0.3) or macroscopic necrosis (*P* = 0.2). Examination of intravoxel heterogeneity via exploring voxel-wise correlations *within* anatomically distinct tumor regions showed similar trends to those observed across patients. Namely, the pooled r-values of voxel-wise sodium and ADC correlations were significantly different from zero and overall positive within areas of suspected NET (Fig. 4A; mean ± standard deviation of r-values = 0.50±0.19, *P* < 0.0001) and CET (Fig. 4B; 0.32±0.29, *P* = 0.0008). Additionally, the voxel-wise correlations of relative sodium concentration and nrCBV were significantly different from zero and overall negative within areas of suspected NET (Fig. 4A; -0.10±0.17, *P* = 0.03) and, interestingly, areas of macroscopic necrosis (Fig. 4C; -0.27±0.21, *P* = 0.005). In NAWM, the voxel-wise correlations of sodium and ADC were significantly different from zero and overall positive (Fig. 4D; 0.25±0.22, *P* = 0.0002), while there was no association between sodium and nrCBV (Fig. 4D; -0.13±0.30, *P* = 0.1).

## Discussion

The current study showed that (1) sodium MR signal was highest in necrotic regions followed by CET and NET; (2) sodium was elevated in CET compared to NET, but not ADC; (3) sodium and ADC were positively correlated in enhancing and non-enhancing tumor subregions, as well as NAWM, but not in necrotic areas; and (4) sodium and nrCBV were negatively correlated in non-enhancing tumor and areas of macroscopic necrosis. These findings add to the previous literature by utilizing sodium MRI together with complementary proton MRI in human gliomas, which allows for further insights into the potential biological underpinnings of sodium imaging as a biomarker.

Our observation that sodium concentration was highest in areas of necrosis was consistent with prior findings by Regnery et al. [23] and can be explained by increased extracellular fluid with high sodium concentration in necrotic tissues and suggests elevated sodium MR signal may have a potential clinical use in identifying treatment-related changes, pseudoprogression or radiation necrosis. Interestingly, relative sodium concentration and proton ADC were positively correlated in NET, CET, and NAWM, but not in necrotic regions. While ADC can be both high or low within areas of necrosis due to heterogeneous extracellular matrix composition and few intact cells restricting free water mobility [8], sodium is likely to be exclusively related to extracellular water concentration as sodium concentration is highly regulated and water molecules form a hydration sphere around sodium ions in solution.

The present study also observed elevated sodium in CET compared to NET, but no difference in ADC. The sodium MR findings are also consistent with the results reported by Regnery et al. [23]. Since extracellular sodium concentration is at equilibrium with plasma and the extracellular volume fraction as measured by ADC can be comparable between CET and NET, these results suggest this increase in sodium concentration may be due to intracellular contribution from an altered metabolic state within enhancing tissue. This appears consistent with the preclinical work by Schepkin et al. [30] who observed elevated sodium MR signal without alterations in proton ADC during tumor recurrence and studies that have observed an increase in intracellular sodium with increasing tumor cell proliferation due to abnormal Na^+^/K^+^-ATPase and NHE activity in glioma cells [17–19]. Additionally, some clinical cases demonstrated hotspots of elevated sodium MR signal not represented by other proton MRI sequences as illustrated in the two representative cases, further suggesting that sodium MRI may be useful to investigate the chemistry of the tumor microenvironment.

The current study also noted higher sodium MR signal and proton ADC within T2 hyperintense regions in treated tumors compared to treatment-naïve tumors. Radiation therapy is known to impact the extracellular matrix by increasing vascular permeability, which in turn would cause increased extracellular fluid that may explain the elevated sodium and ADC values in our treated cohort [38]. These findings add to the growing literature of evaluating treatment response of brain tumors through elevated sodium levels [26–30], though these results may be interpreted with caution given the small sample sizes. Interestingly, even though increased sodium in tumor regions has been speculated to be related to increased angiogenesis that leads to increased extracellular volume fraction [21], the present study observed a *negative* correlation between nrCBV and sodium in NET regions and no association in CET regions, contrary to our initial hypothesis. We speculate that this finding may be explained by a combination of factors including higher angiogenesis leading to reduced extracellular space and while brain edema can cause acidosis and vasodilation, the increased tissue pressure can counteract the vasodilation and lead to reduced perfusion [39]. Further studies with a larger sample size and histological analyses are warranted to better explore these associations.

### Limitations

There are several limitations that should be addressed. First, the current study had a limited sample size and the patients enrolled were heterogenous. Previous sodium MRI studies exploring IDH-status differentiation observed higher sodium in IDH-mut gliomas compared to IDH-wt, but these studies mostly involved untreated tumors [23, 24, 40, 41]. Most of the present study cohort had received prior chemoradiation and very few were IDH mutants, so IDH-differentiation based on sodium MR signal intensity was not explored. Future studies involving a larger study cohort may be valuable to further validate our findings and to assess potential specific associations between sodium MRI and quantitative proton MRI based on IDH-mutational status. Furthermore, an external sodium phantom was unable to be scanned to quantify sodium concentrations, so instead sodium MR signal intensity was normalized to the vitreous humor as done in a prior study [36], though sodium concentration of the vitreous humor measured using sodium MRI may slightly vary in patients with brain tumors [21]. Another potential limitation in the current study was the presence of partial volume contamination from the large sodium MRI voxels [23], which may have influenced the accuracy of sodium MR measurements in smaller tumor subregions. Similarly, partial volume effects of perfusion MRI may have hindered our analyses of suspected necrosis because some necrotic regions appeared to have higher than expected perfusion for dead tissue. Lastly, additional inversion-recovery prepared sodium images, potentially allowing to disentangle intracellular and extracellular sodium contributions [25, 40], could not be acquired due to signal-to-noise ratio and total exam duration limitations. Future studies involving advanced sodium MRI methods [24, 25, 40] in combination with proton MRI may be valuable for assessing gliomas.

## Conclusion

Associations between sodium, ADC, and nrCBV may reflect alterations in extracellular space, tumor proliferation, and angiogenesis. In gliomas, sodium MR signal may reflect a combination of the amplitude of the extracellular space and the increased intracellular sodium concentration. Multi-nuclear MRI may be useful to characterize the brain tumor microenvironment.

## Electronic supplementary material

Below is the link to the electronic supplementary material.

Supplementary Material 1

## Abbreviations

ADC: Apparent diffusion coefficient
ATP: Adenosine triphosphate
CET: Contrast-enhancing tumor
CNS: Central nervous system
GBM: Glioblastoma
IDH-mut: Isocitrate dehydrogenase mutant
IDH-wt: Isocitrate dehydrogenase wild-type
NAWM: Normal-appearing white matter
NET: Non-enhancing tumor
NHE: Na^+^/H^+^-exchanger
VEGF: Vascular endothelial growth factor

## Declarations

### Prior presentation

A portion of the results were presented at the Society for Neuro-Oncology 2022 Annual Meeting.
