# Supplementary material for: Multi-nuclear sodium, diffusion, and perfusion MRI in human gliomas
Source: J Neurooncol. 2023 Jun 9;163(2):417–27. doi: 10.1007/s11060-023-04363-x (PMC10322966; doi:10.1007/s11060-023-04363-x)

Multi-nuclear sodium, diffusion, and perfusion MRI in human gliomas

(Supplement for Journal of Neuro-oncology)

Supplementary Figure Legends

Supplementary Figure 1. Differences in sodium, ADC, and nrCBV based on tumor subregion in all enhancing tumors including without necrosis. Associations remained consistent with results involving only enhancing tumors with necrosis in the main manuscript text. Sodium was significantly higher in CET compared to NET (P=0.0006) **(A)** while there was no significant difference in ADC (P=0.08) **(B)**. nrCBV was also significantly higher in CET compared to NET (P<0.0001) **(C)**.

ADC = apparent diffusion coefficient; nrCBV = normalized relative cerebral blood volume; NET = non-enhancing tumor; CET = contrast-enhancing tumor; △ indicates median; *** indicates P < 0.001; **** indicates P < 0.0001

Supplementary Tables

Supplementary Table 1. Detailed patient treatment information

Pt	Age	Sex	Diagnosis	Prior Treatments	NET (n=20)	CET (n=18)	Necrosis (n=13)
**1**	52	M	Grade 4 IDH-wt Glioblastoma	None (biopsy only)	Y	Y	Y
**2**	46	M	Grade 2 IDH-mut Oligodendroglioma	RT, PCV (procarbazine, CCNU, vincristine) for 3 cycles, PC(V) for total 6 cycles (stopped vincristine in cycle 4 because of neuropathy)	Y	Y	Y
**3**	65	F	Grade 4 IDH-wt Glioblastoma	None	Y	Y	Y
**4**	61	M	Grade 4 IDH-wt Glioblastoma	Sx, RT/Ipilimumab/Nivolumab	Y	Y	
**5**	46	F	Grade 4 IDH-wt Glioblastoma	None	Y	Y	Y
**6**	27	F	Grade 2 IDH-mut Oligodendroglioma	None	Y		
**7**	39	M	Grade 3 IDH-mut Astrocytoma	Sx x2, RT/TMZ, 12 cycles of adj TMZ	Y	Y	Y
**8**	44	M	Grade 4 IDH-wt Glioblastoma	Sx, RT/TMZ	Y	Y	Y
**9**	37	M	Grade 4 IDH-wt Glioblastoma	Sx, RT/TMZ, 6 cycles of adj TMZ	Y	Y	
**10**	60	M	Grade 4 IDH-wt Glioblastoma	Sx, RT/TMZ, 8 cycles of adj TMZ, optune, bevacizumab	Y	Y	Y
**11**	35	F	Grade 4 IDH-wt Glioblastoma	None	Y		
**12**	37	M	Grade 4 IDH-mut Astrocytoma	Sx, RT/TMZ, 4 cycles of adj TMZ, Sx, 5 cycles of adj TMZ	Y	Y	Y
**13**	57	F	Grade 4 IDH-wt Glioblastoma	Sx, RT/TMZ, 4 cycles of adj TMZ	Y	Y	Y
**14**	66	M	Grade 4 IDH-wt Glioblastoma	Sx, RT/TMZ, 12 cycles of adj TMZ/Optune	Y	Y	Y
**15**	70	M	Grade 4 IDH-wt Glioblastoma	Sx, RT/TMZ, regorafenib	Y	Y	
**16**	57	M	Grade 4 IDH-wt Glioblastoma	Sx, RT/TMZ, SRS, 6 cycles of adj TMZ	Y	Y	Y
**17**	58	M	Grade 4 IDH-wt Glioblastoma	Sx, RT/TMZ, 3 cycles of adj TMZ, Icapamespib	Y	Y	
**18**	52	M	Grade 4 IDH-wt Glioblastoma	Sx, RT/TMZ, 8 cycles of adj TMZ	Y	Y	
**19**	55	M	Grade 4 IDH-wt Glioblastoma	Sx, RT/TMZ, 6 cycles of adj TMZ, then 100mg daily for ~2 weeks	Y	Y	Y
**20**	22	F	Grade 4 IDH-wt Glioblastoma	Sx, RT/TMZ, 12 cycles of adj TMZ	Y	Y	Y

Y (Yes) indicates included in analysis

Supplementary Figures

Supplementary Figure 1

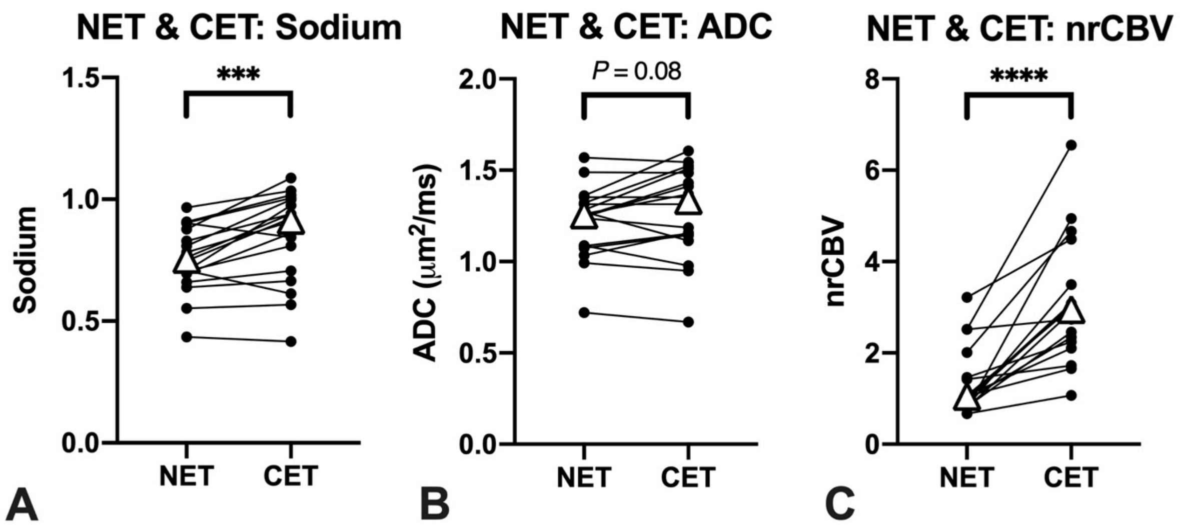

Supplement: Supplementary file 1 — Supplementary Material 1 [file 11060_2023_4363_MOESM1_ESM.pdf]
